# Cesarean Delivery with External Negative Pressure Dressing System: A Retrospective Cohort Study

**DOI:** 10.1055/s-0036-1585470

**Published:** 2016-07-20

**Authors:** Teresa A. Orth, Mary M. Gerkovich, Erica Heitmann, Jonnie Overcash, Charles Gibbs, Marc Parrish

**Affiliations:** 1 Division of Maternal Fetal Medicine, Obstetrix Medical Group of Arizona, Tucson, Arizona; 2 Department of Biomedical and Health Informatics, University of Missouri Kansas City, Kansas City, Missouri; 3 Labor and Delivery, St. Luke's Hospital, Kansas City, Missouri; 4 Division of Maternal Fetal Medicine, University of Kansas Medical Center, Kansas City, Kansas

**Keywords:** negative pressure, wound therapy, cesarean delivery, cesarean section

## Abstract

**Objective** To determine whether the use of external negative pressure dressing system (ENPDS) can reduce the incidence of wound complications after cesarean delivery (CD) compared with traditional dressings.

**Methods** Retrospective review of all patients undergoing CD between November 2011 and March 2013. Information was collected on demographics, body mass index (BMI), duration of labor, pre- and postnatal infections, incision and dressing type, and postoperative course. Comparisons were made between traditional dressing and an external negative pressure dressing system.

**Results** Of 970 patients included in the study, wound complications occurred in 50 patients (5.2%). Comparisons of ENPDS ( *n* = 103) and traditional dressing ( *n* = 867) groups revealed higher wound complications for ENPDS with odds ratio (OR) 3.37 and confidence interval (CI) 1.68 to 6.39. ENPDS was more commonly used in patients with BMI > 30 and preexisting diabetes. After controlling for BMI and pregestational diabetes in logistic regression analysis, ENPDS was equivalent to traditional dressing for risk of wound complications with an adjusted OR 2.76 (CI 0.97 to 7.84), with a trend toward more wound complications with ENPDS. Wound separation also tended to be more common in ENPDS group versus traditional dressing with an adjusted OR 2.66 (CI 0.87 to 8.12), although this result did not reach significance.

**Conclusion** ENPDS is equivalent to traditional dressing for preventing wound complications after controlling for the higher-risk population selected for its use. In particular, wound separation appears to occur more frequently in women treated with ENPDS versus traditional dressing and should be regarded as a potential hazard of the system.

The cesarean delivery (CD) rate has increased substantially since 1996 (21%) and now accounts for 32.8% of all births in the United States. 1 Wound complication rates for CD range from 2.5% in low-risk patients to 30% in morbidly obese women. 2 3 Wound complications account for the majority of the morbidity after CD and include infection, seroma, hematoma, and wound separation. The risk factors for wound complications are numerous and include obesity, diabetes, chorioamnionitis, prolonged rupture of membranes, severe anemia, tobacco use, anticoagulation, severe hypertension, preeclampsia, and steroid use. 4 Olsen et al defined further risk factors for wound complications to include the use of staples, induction of labor, labor >12 hours, or subcutaneous hematoma. 5 The Centers for Disease Control defines surgical site infections (SSIs) as those that occur within 30 days after a procedure. 6

Wound complication, even if not accompanied by an infection, is a significant cause of postoperative morbidity following CD. In addition to the increased cost of care, there is the inconvenience of therapy, increased postoperative pain and convalescence, as well as difficulty with activities of daily living. 7 It is logical, then, to employ measures at the time of surgery that may prevent wound complications and to ensure that there is a demonstrable benefit to their use.

Negative pressure wound therapy is thought to improve wound healing by decreasing bacterial load and wound fluids and by increasing blood flow, oxygenation, angiogenesis, and epithelialization. 8 There is a commercially available battery-powered external negative pressure wound dressing system (ENPDS), the Prevena system (Acelity, San Antonio, Texas), available at the study institution for use for up to 7 days. The Prevena system allows for continuous drainage of the wound following primary incisional closure. This system is external to the closed incision and has benefits including ease of use and amenability to outpatient management. Traditional incision dressings consisting of gauze and tape have no additive benefit other than acting as a barrier to the surrounding environment. Both ENPDS and traditional dressings are applied in a sterile fashion after closure of the incision. Traditional dressings are typically removed on postoperative day 1 or 2 per surgeon preference.

Most studies have focused on the use of negative pressure wound therapy in orthopedic or cardiothoracic surgery. 9 10 11 There is a dearth of literature regarding its use in abdominal surgery and its use after CD. 12 13 14 Mark et al demonstrated a small albeit nonsignificant clinical benefit in using ENPDS in 21 obese women undergoing CD with a 0 versus 10% wound complication rate in ENPDS versus traditional dressing groups; however, there were only 5 wound complications in this total cohort of 63 women and historical controls were used. 13 Chaboyer et al demonstrated no significant difference in the use of ENPDS for obese women after CD. 14 The use of ENPDS in abdominal surgery was evaluated in a retrospective study involving 254 patients undergoing open colorectal surgery. 12 Analysis of the data revealed a significant decrease in the rate of SSI in patients with ENPDS compared with traditional dressing (odds ratio [OR] 0.32, *p* < 0.05). However, the baseline risk of SSI in colorectal surgery (29% in control group) is high.

In contrast to the previous studies on ENPDS and CD, 13 14 we used a contemporaneous control group and all women undergoing CD as a comparison for ENPDS use with traditional dressing. Our cohort did not exclude women who were not obese, a subset of women that had not been assessed in either prior study on ENPDS and CD. In addition, the small sample sizes in previous studies will be bolstered by inclusion of more women using ENPDS in this retrospective review. Our objective was to compare wound complications of patients undergoing CD using either ENPDS or traditional dressing. Planned secondary analyses included collection of demographics, cesarean indications, maternal body habitus, and pregnancy complications. Based on previous studies, including nonobstetric studies, it was postulated that ENPDS would reduce wound complications in patients undergoing CD.

## Materials and Methods

Institutional Review Board approval was obtained prior to initiation of the study, and all guidelines were followed to maintain patient privacy and protect patient health information. The retrospective cohort study was conducted according to the STROBE (Strengthening the Reporting of Observational Studies in Epidemiology) checklist. 15

All patients who had a CD at St. Luke's Hospital in Kansas City, Missouri—a tertiary referral center in our region—were tracked as part of an effort to determine the rate of wound complications at our institution; this quality control initiative has been in place since June 2011. All patients received standard antibiotic prophylaxis per protocol based on patient allergies at least 30 to 60 minutes prior to the skin incision. Information is uploaded into a database that records the patient's medical record number, date of procedure, attending physician, and outpatient follow-up between 1 and 6 weeks, with information recorded regarding wound complications. The database reports basic information regarding the wound complication (present, absent), rather than qualitative data (type of complication, severity, sequelae). Using this database, all patients who underwent CD from June 2011 until March of 2013 were identified for retrospective analysis.

We also reviewed the hospital record, which included scanned reports from outpatient visits as part of the computerized chart. This review allowed qualitative data to be assessed for those patients with wound complications diagnosed after discharge and managed as outpatients. All other information was extracted from the patient's available hospital chart (both paper and electronic record). These follow-up procedures allowed for considerable assurance of complete information on wound complications occurring within the institutional system including private and resident physician clinics.

Information collected from the patient's chart included age, ethnicity, weight at admission, height, body mass index (BMI) at admission, admission stay dates, gravidity, parity, and gestational age at time of delivery. Information about the maternal medical history was recorded including presence or absence of pregestational diabetes and treatment, gestational diabetes, chronic hypertension, maternal autoimmune disease, sickle cell or another hemoglobinopathy, admission hemoglobin < 8 g/dL, postoperative hemoglobin < 8 g/dL, long-term steroid use (more than 3 months), use of anticoagulation, and need for transfusion postoperatively. Obstetric information was also recorded including presence of preeclampsia, HELLP syndrome (hemolysis, elevated liver enzymes, low platelet count), labor prior to cesarean, labor length >12 hours, spontaneous labor, preoperative fever, postoperative fever, prolonged rupture of membranes (>18 hours), and preoperative diagnosis of chorioamnionitis. Information about the surgery performed was recorded including the primary surgeon, prior cesarean, type of skin incision, skin closure technique, estimated blood loss, length of surgery, and dressing used (categories included gauze with tape, pressure dressing with additional packing and tape, silver-impregnated dressing, or EPNDS). Dressing type used was determined by surgeon preference alone. No preselection criteria were designated for ENPDS because this study was retrospective and no hospital policy was operational at the time of the study. Postoperative information was also recorded and included length of stay, readmission for wound complications, endomyometritis, wound infection culture (if performed), and the nature of the wound complication to document whether it was a seroma, hematoma, separation, or infection or a combination of these.

Descriptive statistics were used to describe the patient population and outcomes. Missing data analysis variables were excluded from the analysis. Those patients receiving ENPDS were compared with those receiving traditional dressings (which included gauze with tape, pressure dressing with additional packing and tape, and the small number of silver-impregnated dressings [ *n* = 12]) for differences in age, gestational age at delivery, BMI, pregnancy complications (diabetes, preeclampsia, chorioamnionitis), surgeon type, and length of surgical procedure. The data was analyzed using chi-square for categorical data and the independent *t* test for continuous data. Finally, logistic regression was employed to look at the predictive contribution of dressing type on the occurrence of wound complications after controlling for those variables that showed univariate prediction of wound complications, specifically diabetes, tobacco use, obesity, surgeon type, and preeclampsia.

## Results

A total of 970 patient charts from the 22-month period were available for analysis. Complete data was collected on 99.4 to 99.7% of data variables included in this analysis on wound complications. ENPDS was used in 103 patients (10.6%), and 867 patients received a traditional dressing. The characteristics of the study sample and the two dressing groups are shown in Table 1 . The population delivering at this hospital was mostly white (61.8%) and multigravid (69.6%). The groups did differ significantly in age and gestational age at delivery, but the clinical significance was small. The dressing groups did not differ in ethnicity. Patients receiving ENPDS were significantly more likely to be obese (93%) compared with those who received traditional dressing (61%, *p* < 0.001; see Table 1 ). Those receiving ENPDS were also significantly more likely to have pregestational diabetes (18 versus 3.4%, *p* < 0.001; see Table 1 ). The ENPDS group also had a significantly longer mean surgical time (52.3 versus 60.6 minutes, *p* < 0.001). maternal–fetal medicine physicians were significantly more likely to use the ENPDS than resident clinic physicians, and the private physicians were the least likely to use the ENPDS (21.5% for maternal–fetal medicine, 10.8% for clinic, 4.0% for private, *p* < 0.001; data not shown).

**Table 1:** Demographics comparing ENPDS with traditional dressing groups

	Traditional dressing ( *n* = 867)	ENPDS ( *n* = 103)	*p* Value a
Ethnicity			
Caucasian	538 (62%)	60 (59%)	0.289
African American	171 (20%)	27 (26%)	
Hispanic	110 (13%)	13 (13%)	
Asian	22 (3%)	0 (0%)	
Other	24 (3%)	2 (2%)	
Overall	865 (100%)	102 (100%)	
Age (mean ± SD)	29.3 ± 6	31.0 ± 6	0.012 b
Length of surgery (mean ± SD)	52.3 ± 19	60.6 ± 21	<0.001 b
Gestational age at delivery (mean ± SD)	37.6 ± 4	36.2 ± 5	0.001 b
BMI (mean)	32.4 ± 6	43.3 ± 9	<0.001 b
BMI categories			
<25	75 (9%)	2 (2%)	<0.001
25–29.9	265 (31%)	5 (5%)	
≥30	523 (61%)	95 (93%)	
Overall	863 (100%)	102 (100%)	
Tobacco use	94 (11%)	6 (6%)	0.398
Pregestational diabetes	29 (3%)	23 (18%)	<0.001
Preeclampsia	94 (11%)	31 (30%)	<0.001

Abbreviations: BMI, body mass index (kg/m ^2^ ); ENPDS, external negative pressure dressing system; SD, standard deviation.

a Data analyzed with chi-square test, except where indicated.

b Data analyzed with independent groups *t* test.

**Fig. 1:**
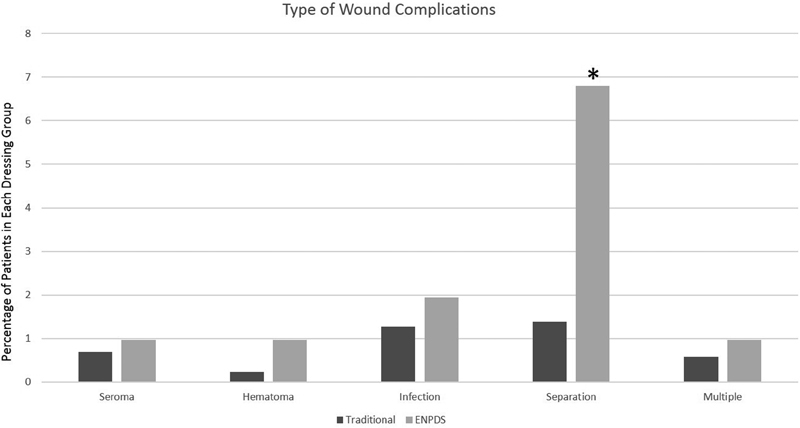
Type of wound complications in traditional versus external negative pressure dressing system (ENPDS). Percentage of patients in each dressing group with wound complications in traditional ( *n* = 870) versus ENPDS ( *n* = 103). Wound complications occurred in 50 patients (5.2%) in this study. Overall wound complications were more common in the ENPDS group. *Significant difference ( *p* < 0.001) in wound separation rate between groups.

Wound complications occurred in 50 patients (5.2%). Wound complications occurred more often with ENPDS than with traditional dressing (ENPDS 12.8%, traditional 4.3%, *p* < 0.05; see Table 2 ). Fig. 1 compares the categories of wound complications per group. The wound infection rates were similar between the groups (ENPDS 1.9%, traditional 1.3%) and not statistically significant ( *p* = 0.49). The patients with ENPDS had a higher rate of wound separation (8.8%) than did the patients with traditional dressing (2.1%; *p* < 0.001). Logistic regression was performed to determine if the high rate of wound separations was more common in ENPDS after controlling for predictor variables in the model. Due to the small number of wound separations ( *n* = 27), this analysis was considered exploratory and revealed a trend for increased rate of wound separation in ENPDS of 2.66 (confidence interval [CI] 0.87 to 8.12), although this result was not significant. Further subgroup analysis of wound complication types was limited by the small sample sizes in other groups.

**Table 2:** Wound complications among dressing groups

Dressing	Wound complication		No wound complication	
	*n*	%	*n*	%
Traditional	37	4.3	829	95.7
ENPDS	13	12.8	89	87.3
Total	50		918	

Note: *p* < 0.05 (significant difference). Missing data of *n* = 1 for follow-up in ENPDS group and *n* = 1 for follow-up in traditional group.

Abbreviation: ENPDS, external negative pressure dressing system.

A series of preliminary unadjusted analyses were conducted to see which variables differed between the traditional and ENPDS groups. Unadjusted, the use of the ENPDS was the strongest predictor of wound complications (OR 3.27, 95% CI 1.68 to 6.39; see Table 3 ). Maternal obesity had a trend toward association with wound complications but was not statistically significant (OR 0.96, 95% CI 0.62 to 1.48), and pregestational diabetes was strongly associated with wound complications (OR 3.16, 95% CI 1.34 to 7.40; see Table 3 ). In addition, primary surgeon type was significantly associated with rate of wound complications (resident clinic service [10.5%] versus maternal–fetal medicine service [4.9%] versus private physicians [1.6%]; Table 3 ).

**Table 3:** Risk factors for wound complications (unadjusted)

Risk factor	Odds ratio	95% CI	*p* Value
ENDPS	3.27	1.68–6.39	0.001
BMI > 30	0.96	0.62–1.48	0.83
Pregestational DM	3.16	1.34–7.40	0.008
Tobacco use	1.47	0.64–3.37	0.36
Preeclampsia	1.35	0.91–1.99	0.13
MFM surgeon compared with clinic resident surgeon	0.44	0.22–0.87	0.018
Private surgeon compared with clinic resident surgeon	0.14	0.06–0.33	<0.001

Abbreviations: BMI, body mass index; CI, confidence interval; DM, diabetes mellitus; ENPDS, external negative pressure dressing system; MFM, maternal–fetal medicine.

An adjusted logistic regression analysis was performed to test whether the dressing type was still a significant predictor when the other variables were controlled. After controlling for BMI (changed to a dichotomous variable with BMI < 30 as the cutpoint) and pregestational diabetes, logistic regression analysis indicated that the risk of wound complication was not significantly different with ENPDS compared with traditional dressing (OR 1.70, 95% CI 0.76 to 3.84). When also including tobacco use, surgeon type, and preeclampsia in logistic regression analysis, ENPDS was still no different from traditional dressing for the risk of wound complications (adjusted OR 1.45, 95% CI, 0.60 to 3.48; see Table 4 ). On combined logistic regression analysis, complete data variables were available for 94.9% of cases.

**Table 4:** Risk factors for wound complication, adjusted (logistic regression)

Risk factor	Adjusted odds ratio	95% CI	*p* Value
ENDPS	1.45	0.60–3.48	0.41
BMI > 30	1.04	1.00–1.09	<0.05
Pregestational DM	2.01	0.77–5.20	0.15
Tobacco use	1.36	0.57–3.23	0.49
Preeclampsia	2.25	1.06–4.78	<0.05
MFM surgeon compared with clinic resident surgeon	0.33	0.15–0.74	<0.05
Private surgeon compared with clinic resident surgeon	0.18	0.08–0.42	<0.05

Abbreviations: BMI, body mass index; CI, confidence interval; DM, diabetes mellitus; ENPDS, external negative pressure dressing system; MFM, maternal–fetal medicine.

## Discussion

During the 22-month study period, the ENPDS was used frequently (103 patients, 10.6% of all CD) and was associated with an increase in wound complication rates when compared with traditional dressing. However, patients receiving ENPDS were selected by surgeons specifically because of comorbid conditions associated with wound complications leading to selection bias, which was controlled for with logistic regression. In fact, all the patients receiving the ENPDS had at least one comorbid condition, most commonly obesity (93%) and diabetes (18%). Because these patients have an inherent increased risk for wound complication, the association between wound complications and the use of ENPDS is likely attributable to patient-level factors that were considered when the provider decided on the use of ENPDS. In fact, when controlling for BMI and pregestational diabetes, the increase in the wound complication rates seen in the ENPDS group disappeared, with the final analysis showing no statistically significant difference between the two dressing groups. For comparison and control analysis, the traditional dressing group had a large percentage of obese patients (523 patients, 61%) and diabetic patients (29 patients, 3%). The longer surgical time in the ENPDS group (8-minute increase) is likely due to the application of the device and is consistent with longer mean surgical times previously reported of 8 to 12 minutes, 13 14 and therefore it was not included in the subgroup analysis. Controlling for these particular classifications did not demonstrate statistically significant differences in the wound complication rates ( Table 1 ), which led us to conclude that even in high-risk patients, the use of the ENPDS does not appear to significantly improve wound complication rates when compared with the use of traditional dressing.

There were large differences in surgeon preference for use of the ENPDS after CD, with maternal–fetal medicine subspecialists the most likely to use the ENPDS (21.5% of patients). There were also large differences in wound complication rates between the surgeon groups, with patients of resident clinic physicians having the highest rates of wound complications (10.5% of patients). However, controlling for these differences did not demonstrate a benefit to using the ENPDS compared with traditional dressing.

Currently, there are three small randomized clinical trials (RCTs) with ENPDS and CD with conflicting results. In one RCT terminated early in 2014 due to slow recruitment, Stitley selected patients based on weight greater than 199 pounds and subcutaneous layer more than 4 cm. Wound complications were seen in 15/26 (58%) patients having ENPDS compared with 10/23 (43%) patients having traditional dressing (see ClinicalTrials.gov identifier NCT00654641). In another pilot study in 2014 from Australia, Chaboyer et al selected patients with a prepregnancy BMI more than 30 and a scheduled repeat cesarean section, which demonstrated no difference in wound complications between the groups (14/44 [32%] in ENPDS versus 17/43 [40%] in traditional, *p* = 0.454). 14 Finally, Heine in 2014 demonstrated a benefit of ENPDS in preventing wound complications after CD. The patients were selected based on a BMI greater than 35 up to 42 days prior to surgery, scheduled CD, and planned subcuticular suture closure. This study demonstrated 16% wound complications in traditional versus 5% in Prevena (7/43 traditional and 2/39 Prevena, no statistical analysis performed; ClinicalTrials.gov identifier NCT01450631). There are also three RCTs actively recruiting patients for ENPDS and CD (ClinicalTrials.gov).

In contrast to the retrospective study by Mark et al, 13 we did not demonstrate an improvement in wound complications in women with CD with the use of ENPDS versus traditional dressing. Some explanations for this difference may include the small ENPDS sample size ( *n* = 21) in the prior study, the higher rate of background wound complications within the control group (10%), inclusion criteria of women with BMI > 45 but no other prespecified risk factors, and the use of historical comparison group. 13 In the current study, there was a larger ENPDS sample size ( *n* = 103), a lower background wound complication rate in our control group (4.25%), and the global inclusion of all BMI categories and comorbid conditions. In addition, we used a contemporaneous control group. All of these factors likely led to a significant difference in patient populations that explain the discrepancies in conclusions of the two studies.

Strengths of this study were the large sample size and relatively complete cohort information (99.4% for individual variables and 94.9% for logistic regression), which allowed for subgroup analysis and controlling for risk factors. Due to the quality initiatives at our hospital, information on all patients undergoing CD is captured at delivery and in postpartum chart review/office communication for wound complications. BMI was calculated from delivery admission height and weight, which improves accuracy of true risk of SSI instead of prepregnancy BMI. As a result, patients at risk could be identified and scrutinized using multiple comparisons. All patients were included and analyzed to reduce and/or control for selection bias. In particular, tobacco use at admission was a significant contributor to the risk of wound complications in our retrospective study and was able to be easily controlled for in our analysis due to inclusion of sufficient controls with the comorbid condition. In addition, this study is the first, to our knowledge, to demonstrate a potential increased risk of wound separation with the ENPDS, and this complication should be considered in future RCT study design.

Despite the large number of patients and the ability to make multiple comparisons, there were also limitations to this study, which include its retrospective nature. There were relatively few overall wound complications (50/970; 5.2%); however, this amount is still the largest number of wound complications reported in a single study in the current literature on CD and ENPDS. Reporting these wound complications and study findings should aid clinicians making daily decisions about wound therapy for CD. Although the benefit is controversial, a post hoc power analysis demonstrated 74% power, which may indicate there were slightly too few patients to demonstrate a difference in the outcome of the groups after controlling for the high-risk population. Although it was assumed that surgeons placed the ENPDS in accordance with the manufacturer's directions, this assumption was not verified. During the study period, no patient selection criteria had been established for the use of the ENPDS at the institution, with the choice of dressing left entirely to the preference of the surgeon.

Prospectively, ENPDS should be restricted to patients at greater risk for wound complications; however, our study would indicate obesity is not the sole risk factor for future study design. Indeed, a combination of risk factors (smoking, diabetes, preeclampsia, among others) might be higher risk than class 1 obesity in women without comorbidities. An estimate of the power analysis for an RCT for 80% power with similar characteristics as our population and use of ENPDS would include 119 experimental subjects (ENPDS) and 872 traditional dressing patients (21 more patients than the current study), or for 90% power would include 153 experimental subjects and 1,036 traditional dressing patients (219 more patients than the current study). None of the currently recruiting RCTs has a goal for recruitment of more than 1,000 total patients (Clinicaltrials.gov).

In conclusion, further investigation is needed prior to a recommendation for or against the routine use of ENPDS in patients (regardless of risk factors) who undergo a CD. Even in patients with substantial comorbid risk factors in this study, ENPDS did not afford a significant advantage in the prevention of wound complications over traditional dressing types. There is also a trend toward increased wound complications, in particular wound separation, in patients who received ENPDS, which may be a potential hazard of the system.
